# The Role of m6A Modification in the Development of Systemic Lupus Erythematosus via the TGF‐β Signaling Pathway

**DOI:** 10.1155/jimr/7142984

**Published:** 2026-09-29

**Authors:** Xin Xia, Rui Qu

**Affiliations:** ^1^ School of Medicine, Jiangsu University, Zhenjiang 212013, China, ujs.edu.cn; ^2^ Faculty of Civil Engineering and Mechanics, Jiangsu University, Zhenjiang 212013, China, ujs.edu.cn

**Keywords:** EMT, fibrosis, m6A, SLE, TGF-β

## Abstract

N6‐methyladenosine (m6A) modification is the most widespread RNA internal modification involved in RNA metabolism, participating in cell biology and immune responses. In the early stage of systemic lupus erythematosus (SLE), m6A modification can affect the production of autoantibodies by regulating the differentiation of T follicular helper (Tfh) cells and the generation of autoreactive B cells. With the development of SLE, its subsequent action in immune complexes (ICs)‐deposited tissues is the promotion of excessive fibrosis, causing progressive organ damage. TGF‐β is the central driver activating fibroblasts, inducing epithelial‐to‐mesenchymal transition (EMT), and promoting extensive extracellular matrix (ECM) deposition via the SMAD pathway, ultimately leading to tissue fibrosis and organ damage. m6A regulators can act on both upstream and downstream components of the TGF‐β signaling pathway. They can synergize with TGF‐β transcription factors to control the amplification of TGF‐β signaling. Furthermore, they can work with target molecules downstream of the TGF‐β receptor (TGF‐βR) to tune TGF‐β/SMAD pathway activity, increasing the expression of profibrotic transcripts. It is suggested that inhibition of m6A modification targeting the TGF‐β signaling pathway can effectively reduce EMT and fibrosis in multiple organs, especially in the kidney, improving the long‐term prognosis of SLE patients. In this review, we focus on the role of TGF‐β in the pathogenesis of SLE, the mechanism by which m6A modifications affect the TGF‐β signaling pathway, and its impact on EMT and fibrosis in different organs, trying to provide a new treatment strategy for the control of SLE symptoms.

## 1. Introduction

Systemic lupus erythematosus (SLE) is a multifactorial systemic autoimmune disease, which is characterized by the presence of autoantibodies, with diverse clinical manifestations involving one or more organs. The global estimates of the prevalence of SLE are 43.7/100,000 people. The global estimated prevalence of SLE is 43.7/100,000, and the estimated incidence is 5.14/100,000 person‐years [1]. There are significant regional differences in the prevalence of SLE, with the highest prevalence of SLE in the United Arab Emirates, Barbados, and Brazil. Women are more frequently affected than men across all age groups and ethnicities (female:male≈8.5:1). The incidence rate peaks in the middle of adulthood [2]. SLE patients usually exhibit various manifestations of vasculitis in the skin, kidneys, lungs, central nervous system, gastrointestinal tract, and other sites. Lupus nephritis (LN) occurs in about 40% of SLE patients, mostly within 5 years of diagnosis, and 4.3%–10.1% of patients progress to end‐stage renal disease (ESRD). Renal failure, along with infections, cancer, and cardiovascular events, is one of the most common causes of death in SLE patients [3]. Based on the International Society of Nephrology/Renal Pathology Society (ISN/RPS) 2003 classification of LN and the 2018 revision, long‐term recurrent episodes of fibrosis progression from class III focal LN or class IV diffuse LN can lead to continuous fibrosis of the mesentery and interstitium, gradually progressing to segmental sclerosis, and ultimately developing into class VI advanced sclerosing LN [4]. The management of SLE aims to prevent progression of chronic kidney disease and flares, address comorbidities, and improve health‐related quality of life [5].

N6‐methyladenosine (m6A) modification is a methylation at the N6 site of adenosine on RNA molecules, which is the most widespread post‐transcriptional RNA modification [6–9]. The process of m6A modification is reversible and dynamic. It is regulated by three types of proteins: writers, erasers, and readers. The function of m6A modification in regulating mRNA metabolism can be summarized as translation, decay, splicing, and nuclear export, thereby affecting cell biology and immune responses. m6A modifications can alter the differentiation and maturation of immune cells, thus influencing the pathogenesis of autoimmune diseases. SLE patients have aberrant m6A modification patterns and altered expression of m6A‐related proteins [10–12]. Previous studies have confirmed the role of m6A modifications in the homeostasis of immune cells in the occurrence and development of SLE [13]. Recent studies elucidate that the m6A changes in autoimmune diseases are involved in TGF‐β signaling, further showing the role of m6A modifications in the development and prognosis of SLE [14]. In this review, we focus on the role of TGF‐β in the pathogenesis of SLE, the mechanism by which m6A modifications affect the TGF‐β signaling pathway, and its impact on epithelial‐to‐mesenchymal transition (EMT) and fibrosis in different organs, providing a new treatment strategy for the control of SLE symptoms and progression.

## 2. Overview of m6A Modification

m6A modification has three types of regulators: writers, erasers, and readers. Writers are methyltransferase complexes, which are responsible for installing m6A modifications at specific sites in target mRNAs. METTL3 (also called MT‐A70) acts as the active catalytic component. METTL14 usually functions synergistically with METTL3 by forming crystal structures [15]. In most cases, METTL3 cooperates with METTL14 and other auxiliary subunits, such as WTAP [7, 16, 17], ZC3H13 [17–19], VIRMA [17, 19], RBM15/15B [17], CBLL1 [17], and KIAA1429 [20, 21], facilitating their localization to the nucleus and m6A methyltransferase activity. The most common writers are METTL3/METTL14/WTAP complexes. Erasers are demethylases, which can remove the methyl group, including ALKBH5 [22–24] and FTO [25, 26]. Readers are RNA‐binding proteins, which can recognize and bind to modified sites directly or indirectly [27]. Readers contain YTH‐domain family 1‐3 (YTHDF1‐3) [28], YTH domain containing protein 1‐2 (YTHDC1‐2) [29], heterogeneous nuclear ribonucleoprotein (HNRNPF, HNRNPG, HNRNPC, and HNRNPA2B1) [30], insulin‐like growth factor 2 mRNA‐binding protein 1‐3 (IGF2BP1‐3) [31], and eIF3 [32]. Through the individual or synergistic effects of m6A regulators, RNA translation, decay, splicing, and nuclear export are affected.

It is well established that m6A regulators can regulate T‐cell differentiation and germinal center (GC) responses. METTL3, together with METTL14, can maintain the stability of *SOCS* mRNA in CD4^+^ T cells, regulating the differentiation of Th cells by the SOCS/STAT5/IL‐7 pathway [33]. METTL3 could promote *SOCS* mRNA translation in regulatory T (Treg) cells to activate the IL‐2/STAT5 signaling axis to enhance the Treg suppressive function [34]. METTL3 can also regulate the translation process of the T follicular helper (Tfh) signature gene *TCF7* in CD4^+^ T cells to control Tfh differentiation [35]. METTL14 can support Treg cell differentiation and their suppressive function through promoting the translation of *RORC* mRNA in CD4^+^ T cells [36]. It is suggested that m6A regulators play an important role in regulating T‐cell differentiation, especially Treg and Tfh cells.

It is proposed that GC responses depend on m6A modification. GC maintenance and selection of high‐affinity clones by T cells rely on MYC functions and activation of its downstream gene transcription. After being activated by extracellular CD40 signaling, the level of IGF2BP3 increases in B cells. METTL3 and IGF2BP3 maintain the stability of *MYC* mRNA, which is suggested to be a constitutive process in B cell, required at the early and late stages of GC responses [37]. Besides, YTHDF2 has been reported to support the proliferation of GC B cells. ALKBH5 can decrease the mRNA stability and protein levels of IFN‐γ and CXCL2, suppressing CD4^+^ T‐cell responses [38]. It is shown that m6A can regulate GC responses by affecting GC maintenance, B‐cell proliferation, and CD4^+^ T‐cell responses.

## 3. Role of TGF‐β in the Pathogenesis of SLE

SLE is characterized by the autoantibodies directed against nuclear components, such as nucleosomes and nucleoproteins; these autoantibodies form immune complexes (ICs), causing chronic inflammation. The amplification process of autoantibodies in SLE can be summarized as follows: Primarily, plasmacytoid dendritic cells (pDCs) activated by ICs can secrete type Ⅰ IFN (IFN‐Ⅰ) and IL‐6, which promote the differentiation of autoreactive memory B cells into plasma cells [39]. In this process, IFN‐Ⅰ and other components can also induce myeloid antigen‐presenting cells (APCs) to upregulate the expression of B cell stimulatory cytokines such as BAFF and APRIL, promoting the activation and differentiation of autoreactive B cells [40, 41]. Second, ICs can activate myeloid APCs and drive the development of Tfh cells through inducing the Tfh‐promoting molecules such as IL‐12, IL‐23, and OX40L. Tfh cells are specialized for providing help to B cells, playing a vital role in the GC reaction and selection of high‐affinity B cells [42, 43]. During the process, IFN‐Ⅰ produced by pDCs can also induce myeloid APCs to secrete Tfh‐promoting cytokines. Exaggerated Tfh‐cell generation accompanied by excessive GC formation causes the expansion of autoreactive B‐cell clones and amplifies the production of autoantibodies [44]. The deposition of generated ICs can cause damage to organs and tissues and lead to the further release of large amounts of nuclear components [45].

Recent studies show that TGF‐β is a major driver of the loss of self‐tolerance and uncontrolled autoimmunity characteristic of SLE. Immune cells (such as macrophages and T cells) and damaged epithelial cells (such as renal tubules and alveolar epithelial cells) can secrete TGF‐β. It is well established that TGF‐β is highly expressed in the inflamed tissues in lupus mouse models, glomerulonephritis lesions, and LN [46, 47]. There is a significant increase in IL‐6 and TGF‐β in patients with severe SLE. Patients with high serum creatinine levels have a significant increase in TGF‐β [48]. The serum levels of TGF‐β1 are highly associated with disease‐related manifestations [49]. When studying organ‐specific features, eye and cardiovascular manifestations are positively correlated with the serum levels of TGF‐β1. In contrast, gastrointestinal and musculoskeletal involvement are associated with lower levels of circulating TGF‐β1 [49]. Further studies indicate that TGF‐β can regulate the aberrant Tfh cell response. TGF‐β can collaborate with IL‐12 and IL‐23 to promote activated CD4^+^ T cells to express multiple Tfh molecules such as CXCR5, ICOS, IL‐21, and Bcl‐6 and to downregulate the Bcl‐6 repressor Blimp‐1 to promote the differentiation of human Tfh cells [50]. Therefore, excessive production of IL‐12 and IL‐23 by myeloid APCs, as well as an environment rich in TGF‐β, may lead to abnormal Tfh cell responses in SLE.

In addition, TGF‐β is the central driver triggering excessive extracellular matrix (ECM) deposition and tissue scarring in organ fibrosis, such as renal, pulmonary, hepatic, and cutaneous fibrosis. It is the most potent signal that activates fibroblasts and promotes their secretion of collagen [51, 52]. Fibroblasts are activated and transformed into myofibroblasts, producing a large amount of ECM. In this process, some epithelial cells can transform into fibroblasts, which is also known as EMT [53, 54]. TGF‐β is the master regulator of the EMT. The combination of TGF‐β and TGF‐β receptor (TGF‐βR) on epithelial cells can activate the SMAD signaling pathway, repressing E‐cadherin expression and inducing mesenchymal transcript expression such as αSMA, vimentin, and fibronectin (FN) [55–57]. Then, epithelial cells lose polarity and connectivity and transform into myofibroblasts. The myofibroblasts secrete ECM such as collagen and FN while inhibiting the activity of ECM‐degrading enzymes (such as matrix metalloproteinase), leading to excessive deposition of ECM in tissue interstices and the formation of fibrosis, especially in the kidney and skin. The excessive fibrotic tissues replace normal and functional cells, causing organs to become stiff and structurally distorted. Fibrosis is the main cause of irreversible organ damage and functional failure in SLE, especially in LN, where it is most prominent and fatal [58]. Preventing and inhibiting fibrosis is an important challenge in improving the long‐term prognosis of SLE patients.

## 4. m6A Modification Involved in TGF‐β Signaling

m6A modification acts as a critical post‐transcriptional regulator of the TGF‐β signaling pathway. It can modify the key mRNAs within the pathway by influencing their stability, decay, splicing, and translation, which is essential for cellular responses. m6A regulators can exert effects on both the upstream and downstream of the TGF‐β signaling pathway. They can synergize with TGF‐β transcription factors to control the amplification of TGF‐β signaling. Furthermore, they can influence target molecules downstream of the TGF‐βR, modulate the activity of the TGF‐β/SMAD pathway, and enhance the expression of profibrotic transcripts, which are crucial for fibrosis and EMT. According to the EULAR Recommendations for the Management of SLE [5], common organs affected in SLE include: (1) kidneys, mainly LN, manifesting as proteinuria, hematuria, and renal dysfunction; (2) skin and mucous membranes, manifesting as erythematous discoid lupus and photosensitivity; (3) joints and muscles, manifesting as polyarthritis and myalgia; (4) cardiovascular system, manifesting as pericarditis, endocarditis, pulmonary hypertension, and thrombosis; (5) respiratory system, manifesting as lupus pneumonia and interstitial lung disease; (6) neuropsychiatric system, manifesting as epilepsy, cognitive impairment, and peripheral neuropathy; (7) hematological system, manifesting as hemolytic anemia, lymphadenopathy, and splenomegaly; (8) digestive system, manifesting as mesenteric vasculitis, pancreatitis, and hepatitis. Some case reports indicate that the eyes and liver may also be affected, manifesting as retinal vasculitis [59] and hepatic vasculitis [60]. We conducted a literature search using m6A and TGF‐β as keywords and selected the studies focusing on organs affected by SLE, with research directions on fibrosis or EMT. The following results were obtained and are summarized in Table 1.

**Table 1:** Evidence for the role of m6A modification in TGF‐β‐driven EMT and fibrosis in SLE and related diseases.

Evidence category (source)	Organ/disease context	Model system	Key m6A–TGF‐β finding	References	Relevance to SLE
A. Human SLE/lupus nephritis (LN)	Systemic lupus erythematosus (systemic)	Patient PBMCs/blood cells	Aberrant global m6A modification patterns and altered expression of m6A‐related regulators in SLE patients; m6A‐modified lncRNAs involved in SLE	[10–12]	Direct evidence
	SLE with renal involvement	Patient serum	Significantly increased IL‐6 and TGF‐β in severe SLE; higher TGF‐β in patients with high serum creatinine	[50]	Direct evidence
	SLE organ‐specific manifestations	Patient serum	Serum TGF‐β1 levels correlate with disease‐related manifestations (positively with eye/cardiovascular; negatively with gastrointestinal/musculoskeletal)	[51]	Direct evidence
B. Lupus animal models	Inflamed tissues in lupus	Lupus‐prone mouse models	TGF‐β highly expressed in inflamed tissues of lupus mouse models (autoimmunity‐mediated organ damage)	[48]	Direct evidence
C. Non‐lupus fibrotic/inflammatory models	Skin—systemic sclerosis (SSc)	Human SSc PBMCs; dermal fibroblasts in vitro	m6A hypermethylation of TGF‐β pathway transcripts (TGFβ1, TGFβR2) promotes TGF‐β signaling; METTL3 inhibitor STM2457 suppresses TGF‐β‐driven profibrotic genes (ACTA2, COL1A1)	[14]	Mechanistic hypothesis
	Skin—keloids	Human keloid tissue; keloid fibroblasts (KFs); in vivo model	m6A reader HNRNPC stabilizes WDR77 mRNA, upregulating TGF‐β/SMAD3 and driving keloid progression	[61]	Mechanistic hypothesis
	Kidney—acute kidney injury (AKI); hyperuricemic nephropathy	AKI models; human kidney biopsies; cultured tubular epithelial cells	METTL3 elevated in renal tubules; METTL3/IGF2BP2 modulates TAB3 (stability ↑) and ABCG2 (stability ↓) mRNAs, promoting renal dysfunction and fibrosis; METTL3 inhibition attenuates injury	[62, 63]	Mechanistic hypothesis
	Kidney—injury‐induced fibrosis	Proximal tubule‐specific METTL3 knockout mice	METTL3/IGF2BP3 stabilizes β‐catenin mRNA, inducing profibrotic genes and kidney fibrosis; METTL3 knockout protects against fibrotic lesions after injury	[64]	Mechanistic hypothesis
	Kidney—UUO; TGF‐β1 in vitro	Unilateral ureteral obstruction (UUO) mice; HK‐2 cells	METTL3 inhibition reduces TGF‐β‐induced fibrotic markers and UUO fibrosis; m6A stabilizes TGF‐β‐mediated NET1 mRNA to promote EMT; TGF‐β upregulates METTL3 (feedback loop)	[65]	Mechanistic hypothesis
	Kidney—UUO; TGF‐β1 in vitro	UUO mice; TGF‐β1‐treated cells	FTO (eraser) promotes fibrogenesis by stabilizing SQSTM1 mRNA; FTO silencing alleviates UUO‐ and TGF‐β1‐induced inflammation, apoptosis and impaired autophagy	[66]	Mechanistic hypothesis
	Eye—corneal endothelium (Fuchs endothelial corneal dystrophy)	Corneal endothelial cells; METTL3 intervention	METTL3/YTHDF1 promotes Tgfβ2 mRNA translation, activating TGF‐β signaling and endothelial‐to‐mesenchymal transition; METTL3 inhibition ameliorates this process	[67]	Mechanistic hypothesis
	Eye—retinal pigment epithelium (EMT)	hTERT RPE‐1 cells (TGF‐β2‐treated); conjoint m6A‐Seq/RNA‐Seq	METTL3 knockdown enhances, whereas YTHDF1 knockdown suppresses, TGF‐β2‐induced EMT markers (N‐cadherin, FN, SLUG, vimentin) and cell migration	[68]	Mechanistic hypothesis
	Liver—fibrosis	Bile duct ligation model; human hepatic stellate cell line LX‐2	WTAP stabilizes FBLIM1 mRNA, activating TGF‐β signaling, hepatic stellate cell activation, liver fibrosis and inflammation	[69]	Mechanistic hypothesis
	Kidney—experimental glomerulonephritis	Experimental glomerulonephritis model	Elevated TGF‐β and proteoglycan production, expanding mesangial extracellular matrix (evidence at the TGF‐β level)	[49]	Mechanistic hypothesis
D. Cancer/other disease models	Liver—hepatocellular carcinoma (HCC)	Human HCC tissues; nude mouse xenografts	FTO stabilizes BUB1 mRNA via YTHDF2; BUB1 interacts with TGF‐βR1 to activate TGF‐β signaling, promoting EMT and tumor growth; FTO silencing reverses EMT/tumor growth	[70]	Mechanistic hypothesis
	Pancreatic cancer (EMT)	Pancreatic cancer cells (TGF‐β1‐treated; baicalein)	FTO mediates TGF‐β1‐induced EMT by demethylating ZEB1 mRNA (reduced stability); FTO knockdown reverses fibroblast‐like changes (E‐cadherin ↑, N‐cadherin/vimentin/ZEB1 ↓)	[71]	Mechanistic hypothesis
	Liver—HCC metastasis	HCC cells	METTL3/IGF2BP2 stabilizes IQGAP3 mRNA; IQGAP3 activates TGF‐β/SMAD signaling, driving EMT and a metastatic phenotype	[72]	Mechanistic hypothesis
	Lung—non‐small cell lung cancer (NSCLC)	NSCLC cells	VIRMA (writer) methylates ADAR, enhancing TGF‐β signaling; VIRMA knockdown suppresses growth, migration and invasion	[73]	Mechanistic hypothesis
	Gallbladder carcinoma (EMT/metastasis)	Gallbladder carcinoma cells	TGF‐β1 inhibits ALKBH5 binding to the FOXA1 coding sequence, increasing m6A and reducing FOXA1 translation; FOXA1 suppresses TGF‐β1‐induced migration and EMT	[74]	Mechanistic hypothesis

### 4.1. Skin

Enrichment analysis reveals TGF‐β signaling among the top differentially methylated pathways in systemic sclerosis (SSc) compared to control PBMCs. Further analysis validates the changes in the methylation of the TGF‐β pathway components TGFβ1 and TGFβR2, indicating that m6A modifications are abundant in the key transcripts involved in TGF‐β signaling. METTL3 inhibitor STM2457 ameliorates TGF‐β‐driven profibrotic gene expression, including ACTA2 (αSMA) and COL1A1 in dermal fibroblasts [14]. It is reported that keloids exhibit a hyper‐m6A modification status, and HNRNPC is overexpressed in keloid tissues and keloid fibroblasts (KFs). The knockdown of HNRNPC inhibits the migration and proliferation of KFs and limits keloid progression in vivo. Further experiments identify the WD repeat domain 77 protein (WDR77) as an m6A‐dependent downstream target of HNRNPC. HNRNPC can promote the expression of WDR77 by increasing its mRNA stability, elevating the expression of TGF‐β and SMAD3 [75]. It is suggested that pharmacologic inhibition of m6A modification can reduce skin fibrosis and limit keloid progression in the development of SLE.

### 4.2. Kidney

METTL3 expression is elevated in renal tubules in acute kidney injury (AKI) models, human biopsies, and cultured tubular epithelial cells [76, 61]. Proximal tubule‐specific knockout of METTL3 in mice protects the kidney from developing fibrotic lesions after injury. METTL3 can stabilize β‐catenin mRNA, which can be mediated by IGF2BP3, increasing β‐catenin protein, inducing its downstream profibrotic genes, and promoting kidney fibrosis [62]. Moreover, TGF‐β‐activated kinase 1 binding protein 3 (TAB3) and ABCG2 are identified as targets of METTL3, dependent on IGF2BP2. The binding of METTL3 to their m6A‐modified stop codon regions increases the stability of TAB3 mRNA [61] and decreases the stability of ABCG2 mRNA [76], promoting renal dysfunction and fibrosis. Inhibition of METTL3 in HK‐2 cells decreases TGF‐β‐induced fibrotic marker expression and attenuates the degree of kidney fibrosis in the unilateral ureteral obstruction (UUO) mouse model. NET1 is a specific guanine nucleotide‐exchange factor for RhoA [63]. m6A modification enhances the stability of the TGF‐β‐mediated NET1 mRNA and increases the NET1 mRNA expression. In addition, NET1 knockdown decreases the expression of fibrotic markers, indicating that m6A modification on NET1 mRNA plays a role in TGF‐β signaling and EMT [64]. Further study reveals that FTO can also promote fibrogenesis by regulating the stability of SQSTM1 mRNA. FTO silencing has been shown to alleviate UUO‐ and TGF‐β1‐induced inflammation, apoptosis, and impaired autophagy [77]. In the kidneys, METTL3 and FTO theoretically have opposite functions but exhibit similar promoting effects in fibrosis. It is suggested that the inhibition of m6A regulators needs to be refined at specific locations in the kidney to achieve better antifibrotic effects [76].

### 4.3. Eye

METTL3 can promote the translation of *TGF-β2* mRNA in a YTHDF1‐dependent manner, leading to the upregulated TGF‐β2 protein and activation of TGF‐β signaling. Intervention of METTL3 expression or catalytic activity can suppress TGF‐β signaling activation, ameliorating the corneal endothelial‐to‐mesenchymal transition process (EndMT) [65]. It is observed that 10 core EMT genes *ACTG2*, *BMP6*, *CDH2*, *LOXL2*, *SNAIL1*, *SPARC*, *BMP4*, *EMP3*, *FOXM1*, and *MYC* were evaluated in retinal pigmental epithelium (RPE)‐1 cells treated with TGF‐β2 by conjoint m6A‐Seq/RNA‐Seq analysis. METTL3 knockdown enhances RPE‐1 cell migration and significantly upregulates the EMT markers N‐cadherin (encoded by *CDH2*), FN, Snail family transcription repressor (SLUG), and vimentin. However, YTHDF1 knockdown has the opposite effect and decreases both cell migration and the N‐cadherin, FN, and SLUG expression levels [66]. It shows that METTL3 can regulate TGF‐β‐induced EMT in retinal epithelial cells.

### 4.4. Liver

FTO is significantly upregulated in hepatocellular carcinoma (HCC) tissues compared to normal liver tissues, especially in advanced tumor‐node‐metastasis stages and metastatic HCC. Knockdown of FTO decreases colony formation, invasion, EMT, and tumor growth in nude mice. FTO downregulation results in increased m6A modification of *BUB1* mRNA, enhanced degradation via the YTHDF2‐dependent pathway, and reduced BUB1 protein levels. BUB1 can interact with TGF‐βR1, activating downstream TGF‐β signaling. It means that FTO can increase the stability of *BUB1* mRNA by the YTHDF2‐dependent pathway, activating TGF‐β signaling [67]. FTO knockdown reverses the fibroblast‐like changes induced by TGF‐β1, countering TGF‐β1‐induced EMT decrease in E‐cadherin and increase in N‐cadherin, vimentin, and ZEB1. Bioinformatic analysis further identifies that ZEB1 is an m6A‐modified target regulated by FTO. FTO knockdown increases m6A modification and decreases *ZEB1* mRNA stability, suggesting that FTO plays a mediating role in TGF‐β‐induced EMT [68]. It is also reported that METTL3 catalyzes m6A modification of oncogenic protein IQ motif‐containing GTPase‐activating protein 3 (IQGAP3) mRNA. IGF2BP2 binds m6A‐modified IQGAP3 transcripts to prolong their half‐life. Upregulated IQGAP3 activates TGF‐β/SMAD signaling and drives EMT, thereby promoting a metastatic phenotype [70]. It is reported that filamin‐binding LIM protein 1 (FBLIM1) can promote human hepatic stellate cell (HSC) line LX‐2 activation and fibrosis through the WTAP/FBLIM1/TGF‐β axis. WTAP can stabilize *FBLIM1* mRNA and promote the TGF‐β signaling pathway, leading to liver histopathological injury, liver fibrosis, and an inflammatory response [71]. Overall, m6A modifications can promote the translation of the upstream molecules in TGF‐β signaling, such as BUB1, IQGAP3, and FBLIM1 in the liver, activating TGF‐β signaling.

### 4.5. Lung

VIRMA is overexpressed in non‐small cell lung cancer (NSCLC). VIRMA can modify ADAR through methylation, enhancing the activation of the TGF‐β signaling pathway. Reduction of VIRMA suppresses the growth, migration, and invasion of NSCLC cells [72].

The above evidence suggests the prevailing role of m6A in cellular phenotypic transition and fibrosis, as shown in Figure 1. Understanding the tissue‐dependent role is key to developing effective m6A‐targeting therapies. Systemic inhibition of m6A modification is risky because it could worsen the underlying autoimmunity while potentially reducing fibrosis. Current research focuses on cell‐specific targeting or targeting downstream signaling components by regulating their m6A modification.

**Figure 1:**
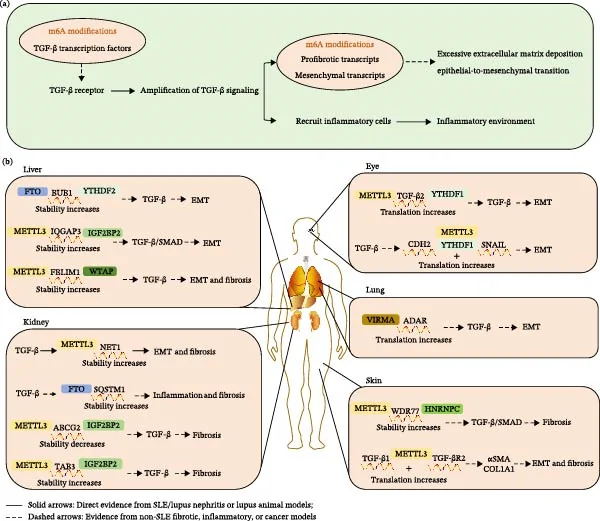
m6A modifications promote EMT and fibrosis by regulating TGF‐β signaling pathway, involving kidney, lung, skin, eye, and liver. (a) The role of m6A modifications in the TGF‐β signaling pathway. (b) The potential relevance of m6A‐mediated TGF‐β signaling in organs affected by SLE.

## 5. m6A Modification Regulated by TGF‐β

m6A modification is not a passive mark but a dynamic and powerful regulator that fine‐tunes the intensity and duration of TGF‐β signaling, with profound implications for development and disease. The regulation creates a dynamic feedback loop in which TGF‐β signaling can, in turn, affect the cellular m6A machinery.

On the one hand, TGF‐β could affect the expression of m6A regulators. Jung et al. found that inhibition of METTL3 in HK‐2 cells decreases the TGF‐β‐induced fibrotic marker expression. Interestingly, TGF‐β stimulation of HK‐2 cells elevates the levels of METTL3 in a dose‐ and time‐dependent manner, along with an increase in the cellular m6A levels [64]. On the other hand, TGF‐β could influence the binding capacity of m6A regulators to their target transcript. Wu et al. confirmed that ALKBH5 promotes FOXA1 protein expression by inhibiting m6A modification on the coding sequence (CDS) region of *FOXA1* mRNA. TGF‐β1 promotes m6A modification by inhibiting the binding capacity of ALKBH5 to the *FOXA1* CDS region, regulating the translation efficiency of *FOXA1* mRNA. Moreover, overexpression of FOXA1 suppresses TGF‐β1‐induced migration and EMT in gallbladder carcinoma cells. It means that TGF‐β1 can regulate EMT and metastasis by inhibiting FOXA1 translation efficiency through m6A modification [69].

## 6. Conclusion and Perspective

In conclusion, m6A modifications work throughout the entire SLE process. In the early stage of SLE, m6A modification can affect the production of autoantibodies by regulating the Tfh‐cell response and the generation of autoreactive B cells. With the development of SLE, its subsequent action in IC‐deposited tissues is the promotion of excessive fibrosis, leading to progressive organ damage. Inhibition of m6A modification targeting the TGF‐β signaling pathway may effectively reduce EMT and fibrosis in multiple organs, especially in the kidney, improving the long‐term prognosis of SLE patients.

Currently, some m6A‐targeting studies in the kidney have made progress. For example, Cpd‐564 and S‐adenosyl‐L‐homocysteine (SAH), which are METTL3 inhibitors, have been shown to have protective effects against cisplatin‐ and ischemia/reperfusion‐induced renal injury and inflammation [61]. Compounds extracted from flavonoids, such as the total flavones of *Abelmoschus manihot* (TFA), ST024026, ST99039, and ST4135876, also show effective function in the inhibition of METTL3. Additional experiments validate that TFA can reduce the inflammasome activation in high‐glucose‐treated MPC‐5 cells through the PTEN/PI3K/Akt pathway [73, 74]. These cases show the potential of m6A‐targeting therapy in treating LN, which also provides clues for the development of effective drugs and informs novel diagnostic strategies to treat SLE.

## Author Contributions

All authors listed have made a substantial, direct, and intellectual contribution to the work.

## Funding

This work was supported by the National Natural Science Foundation of China (Grant 12302012).

## Disclosure

All author have approved the work for publication.

## Conflicts of Interest

The authors declare no conflicts of interest.

## Data Availability

The data supporting the conclusions of this article are included in the article.
